# Enhanced Growth of Multipurpose Calliandra (*Calliandra calothyrsus*) Using Arbuscular Mycorrhiza Fungi in Uganda

**DOI:** 10.1100/2012/830357

**Published:** 2012-12-17

**Authors:** Esther Sebuliba, Phillip Nyeko, Mwanjalolo Majaliwa, Gerald Eilu, Charles Luswata Kizza, Adipala Ekwamu

**Affiliations:** ^1^Department of Forestry, Biodiversity and Tourism, School of Agricultural and Environmental Sciences, Makerere University, P.O. Box 7062, Kampala, Uganda; ^2^Regional Universities Forum for Capacity Building in Agriculture (RUFORUM), Kampala, Uganda

## Abstract

This study was conducted to compare the effect of selected arbuscular mycorrhiza fungi genera and their application rates for enhanced Calliandra growth in Uganda. The performance of Calliandra under different types and rates of arbuscular mycorrhiza fungi inoculation was assessed in the greenhouse using sterilized Mabira soils. Four dominant genera were isolated from the rhizosphere of sorghum in the laboratory. Calliandra seeds were grown in pots and the seed coating method of application was used at concentrations of 0 spores, 30 spores and 50 spores. Each treatment was replicated three times. All Calliandra inoculated seedlings showed improved seedling growth (in terms of height and shoot dry matter weight) compared to the control (*P* < 0.05) except with the arbuscular mycorrhiza fungi mixture treated Calliandra at 50 spores rate. *Glomus* sp. and *Acaulospora* sp. had significant influence on the height of Calliandra, while AMF mixture performed best in terms of shoot dry weight (*P* < 0.05). This study provides a good scope for commercially utilizing the efficient strains of arbuscular mycorrhiza fungi for beneficial effects in the primary establishment of slow growing seedlings ensuring better survival and improved growth.

## 1. Introduction

Arbuscular mycorrhizal fungi (AMF) and legume nodule bacteria, rhizobia are known to improve the ability of plants to uptake and acquire mineral nutrients. AMF is particularly important in nutrient and water uptake from the soil and to improve the plants' resistance to disease [1, 2]. Due to their extramatrical hyphae, mycorrhiza is capable of absorbing and translocating nutrients to the associated plant roots [3]. That way, they increase the supply of slowly diffusing ions, such as phosphates to the plant [4]. Current estimates suggest that 95% of plant species are capable of forming mycorrhiza association [5]. Clearly, mycorrhizal symbiosis is important for plant nutrition [6]. This type of symbiosis is often obligatory to the survival of the host plant. Thus, AMF symbiosis is the subject of much research which has clearly been shown to enhance tree and plant performance [7].

Although multiplication of AMF for increasing plant production is receiving increased attention [8], few studies have been carried out in Uganda. AMF have got broad host ranges whereby some species are more effective with particular host plants in increasing nutrient uptake and plant growth. They therefore differ in the manner and extent to which they colonise roots [9] and also differ in their capacity to form propagules [10]. There is therefore a need to test the performance of trees and crops while in association with different AMF species.

For this study, Calliandra was selected as the host plant mainly because of its fast growth, ease of establishment, and being a legume, it demonstrates the positive features associated with soil improvement [11]. Due to its fast growth, it has found favour in regreening eroded sites, for the smothering of weeds and provision of a light shade for other cash crops. It is an agroforestry tree and its leaves provide valuable fodder for animals. The wood is suitable for fuel wood and it has proved to be a useful alternative to Leucaena species and Eucalyptus. It can be embraced by forest dwellers or agricultural communities. The species belongs to the family Leguminosae and the subfamily Mimosoideae [11]. Hence, Calliandra represents suitable tree species on which to test the best AMF application rate. This study was therefore carried out to determine the best arbuscular mycorrhiza application rate for enhanced Calliandra growth.

## 2. Materials and Methods

The study was conducted in the greenhouse at MAK for Calliandra.

### 2.1. Inoculum Production

Inoculants were produced using the traditional pot culture technique [12]. Soil samples were collected from National Semi Arid Resources Research Institute (NASARRI) in Soroti district, Eastern Uganda to kick start inoculant production for mycorrhiza multiplication and transferred to the Makerere University greenhouse. This was done because preliminary baseline information done under confined field trials for Bt Cotton in Serere revealed that the area yielded high abundance of mycorrhiza spores which would provide the required number needed to form sufficient inoculants. Exposure of the soils to extreme temperatures during transportation was minimized by establishing the bioassay, in the MAK Soil science Department greenhouse, as soon as possible from the field. No additional nutrients were added. Seeds of sorghum were planted into 5 pots carrying 25 kg of the soil sand mixture in the ratio 3 : 2. The sand was treated with 1 M hydrochloric acid for two hours and thoroughly rinsed in distilled water until pH was from 6.8–7. [13]. Eight plants were allowed to grow five weeks with regular watering to field capacity. Four extra weeks were further allowed for hardening off to ensure that harsh conditions prevailed to enhance VAM spore formation [14].

From the greenhouse, after thorough mixing of the soil samples in each pot, 50 g soil samples were extracted and transferred to the botany laboratory at Makerere University (MAK) for spore identification and isolation. Spores of VAM were isolated from soil by the wet sieving and the decanting technique [15]. Preservation of the extracted spores was done using the L-dry or cryopreservation technique [16]. The extracted VAM spores were introduced onto Calliandra seeds using the seed coating—VAM inoculum method. Methyl cellulose was the adhesive used for coating the different inoculants concentrations of 30 and 50 spores each onto the seeds [17]. Soil that had been collected from the different forest regimes of Mabira Forest were thoroughly mixed together for homogeneity and then steam heated. The steam treatment was done in big drums for seven hours to destroy any inherent mycorrhiza spores and corresponding mycoparasites. The inoculated seeds were planted on pre-heated soils to destroy inherent mycorrhizal spores and their corresponding mycoparasites.

### 2.2. Experimental Design

In the greenhouse, the effect of 10 AMF treatments was compared to a control (no AMF) in order to assess their performance on the growth of Calliandra (early phase of growth) using a completely randomized design. Calliandra seeds were obtained from the National Forestry Authority Seed Centre of Uganda. The five treatments included 4 genera types, namely, 6 of *Acaulospora* sp., 10 of *Glomus* sp., 5 of *Gigaspora* sp., and 9 of *Scutellospora *sp., plus their mixture based on the relative abundance from the place of extraction) and two inoculation rates 30 and 50 spores per seed were used in pots of 25 cm depth, and 15 cm diameter, and for 3 kg of sterilized soil.

### 2.3. Height and Biomass Determination

Three randomly selected plots of (2 × 2) m were demarcated in each plot for plant height and dry matter measurements. Height measurements were taken every week after germination for Calliandra. Calliandra was harvested after eighteen weeks and their dry weight determined. The dry weight of the plants from the greenhouse was measured in the Soil Science laboratory of Makerere University. Shoot dry weight of Calliandra was determined by oven drying the plants at 60°C for 24 hours. This was followed by cooling of the plants in a dessicator after which they were weighed on an analytical weighing scale. The Relative Field Mycorrhizal Dependency was computed using Plenchette et al. [18] formula

(1) RFMD={Dry  weight  of  Mycorrhizal  plantDry  weight  of  non-mycorrhizal  plant   −Dry  weight  of  non-mycorrhizal  plantDry  weight  of  non-mycorrhizal  plant}×100.

This formula was used to determine the extent of growth increase for Calliandra due to AMF and ranged between 0% and 100%.

### 2.4. Data Analysis

The height and biomass data was analyzed using ANOVA in Genstat Statistical Package, Discovery Edition version 3. For all variables analyzed using ANOVA, the least significant difference (LSD) in ANOVA post-hoc tests was used to determine differences between genera types, rates of application and weeks after germination. The growth function for Calliandra was determined using linear regression techniques. The inflection points were determined for each treatment by setting the first derivative of the growth function to zero. The relative change in Calliandra height was computed using the following formula:

(2) $$\begin{array}{c} H_{r}=\frac{H_{f}-H_{o}}{H_{o}}, \end{array}$$

where *H*_*r*_ is the relative change in height, *H*_*f*_ is the height of Calliandra at time *t*, and *H*_*o*_ is the height of the control.

## 3. Results

### 3.1. Greenhouse Experiment on Calliandra (*Calliandra calothyrsus*)

#### 3.1.1. Temporal Change in Growth of Calliandra

**Figure 1:**
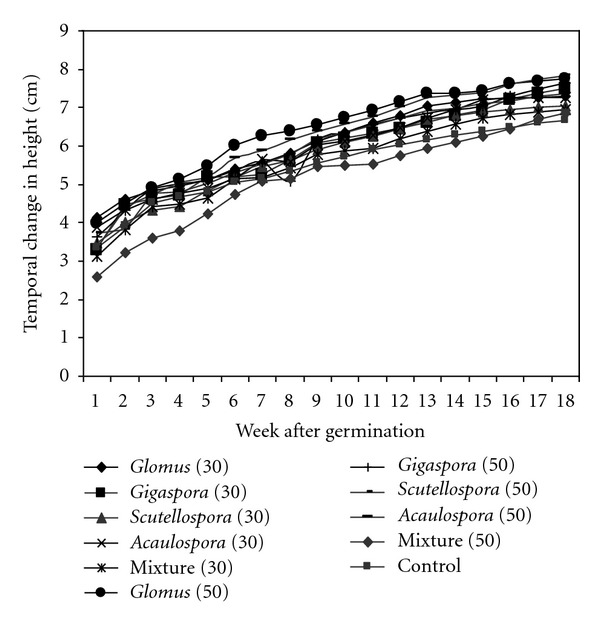
Temporal change in height of Calliandra.

The temporal change in Calliandra height varied significantly within a week, rates of application and genera types (*P* ≤ 0.05). (Figure 1) 50 spores rates of application and with single AMF isolates gave the best results for Calliandra in comparison with the control and 30 spores' rates of application (*P* ≤ 0.05). *Glomus* sp., showed the best results followed by *Acaulospora *sp., *Gigaspora* sp., and then *Scutellospora *sp. Poor results were exhibited by the mixture.The temporal trend in height of Calliandra for the first 18 weeks was natural logarithmic and the regression coefficients ranged from 0.95 to 0.98, the slopes from 1.18 to 1.59, and the intercepts ranged from 2.93 to 3.48.

#### 3.1.2. Relative Change in Height of Calliandra

**Figure 2:**
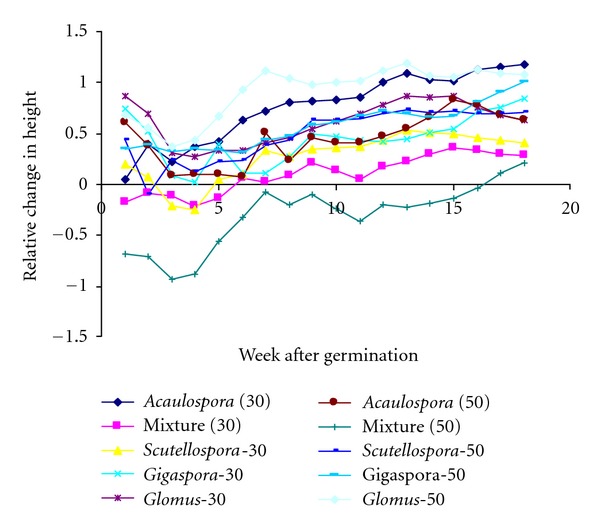
Relative change in height of Calliandra.

The relative change in Calliandra height varied significantly (*P* ≤ 0.05) within a week, rate of application and genera type (Figure 2). Single isolates of AMF and at the 50 spores rates of application showed the best results as compared with the 30 spores rate. *Glomus* sp. gave the best results for relative change in height while the mixture gave the worst results.

For all genera the relative change in height decreased for a few weeks after germination (WAG) and then increased steadily until the 18th WAG. The inflection point was attained after 1.57 WAG for the mixture and 3.95, 3.96, 4.4 to 5.2 WAG for *Acaulospora *sp., *Scutellospora *sp., *Glomus *sp., and *Gigaspora* sp., respectively.

#### 3.1.3. Dry Weight of Calliandra (*Calliandra calothyrsus*)

**Figure 3:**
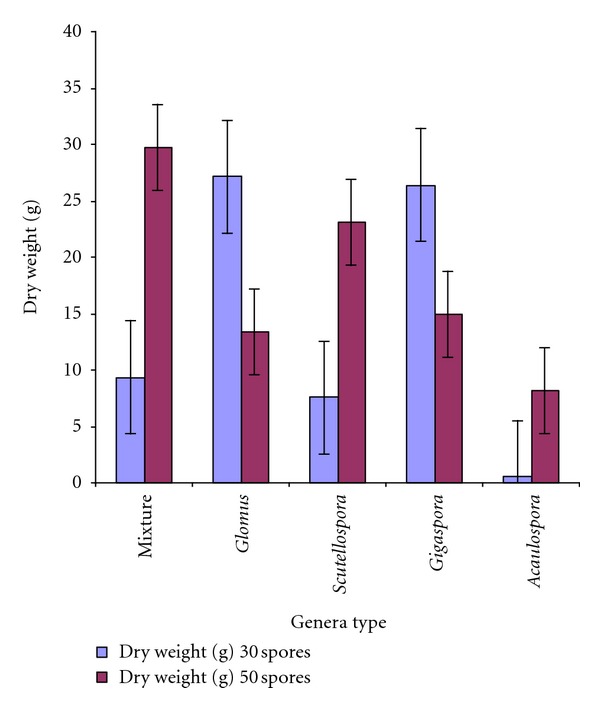
Dry weight of Calliandra at eighteen weeks following inoculation with different mycorrhiza genera.

The mixture of AMF isolates and at the 50 spores application rate gave the best results for dry weight of Calliandra (*P* < 0.05) (Figure 3).

The dry weight of Calliandra varied significantly with rate and genera (*P* < 0.05) and Calliandra dry weight was relatively higher for the 50 spores' rate in comparison to the 30 spores' rate. Considering genera, the mixture 50 had the best dry weight followed by *Scutellospora *sp. 50 and in the third place was all the rest (*Gigaspora* sp. 50, *Glomus* sp. 50, and *Acaulospora *sp. 50). Dry weight of the Calliandra at the 30 spore rate was highest for *Glomus *sp. followed by *Gigaspora *sp. The mixture 30 and *Scutellospora *sp. 30 were significantly lower than the latter and *Acaulospora *sp. 30 had the least dry weight (Figure 3).

#### 3.1.4. Relative Field Mycorrhiza Dependency for Calliandra (*Calliandra calothyrsus*)

The Relative field mycorrhiza dependency (RFMD) for the 50 spores' rate of application was 3.66% greater than that for the 30 spores' rate of application. For the 50 spores rate of application, the mixture had the best RFMD (29.66%) and *Glomus *sp. at 30 spores' rate of application while *Acaulospora* sp. gave the worst results (Table 1).

## 4. Discussion of Results

Inoculated Calliandra plants showed increase in height compared to uninoculated ones. The two genera *Glomus* sp. and *Acaulospora* sp. performed better in enhancing Calliandra height. This could be due to variation in colonization potential of AMF. *Acaulospora* sp. spread hyphae faster in soil [19, 20]. Enhanced height may also be attributed to alterations in the root structural design such as an increase in lateral root development [21–23]. [24] reported that lateral roots are highly colonized and it appears that the symbionts trigger alterations in root structural design to create the most favored sites of interaction.

Although plants treated with a mixture of mycorrhiza had the lowest height, they had the highest dry matter value. The mycorrhiza mixture treated species had the best shoot dry weight and relative field mycorrhiza dependency probably due to their greater root colonization by various AMF species facilitated by a higher extent of host response as also reported by several studies [25–29]. The functional complementarity within the mycorrhiza mixture has been reported to boost productivity even for the most complex AMF community [30–34]. A reduction of AMF biodiversity from four (mixture) to a single AMF taxon resulted into a decrease in biomass of several plant species and in turn a change in plant community structure [35]. 

In another experiment [36], fungal diversity was manipulated and significant increases in nutrient capture, plant diversity, and productivity were found in response to increasing AMF species concentration the case of the 50 spores rate inoculum.

Besides, such wide ranging single or mixture effects of AMF species on several plant variables are also in accordance with previous studies [32, 33, 36] where biomass and nutrient capture of a plant community varies independently with the identity of the inoculated AMF species. Mixtures of AMF have been Curculigo orchioides, an anticancerous herb in India [37] and on frequently used crop species (tomatoes, parsley, pepper, and carrot) in Slovenia [38]. The mixture of AMF has the potential to increase the growth of Calliandra species.

The acid nature of most (over 70%) soils in Uganda [39, 40] and the corresponding phosphorus deficiency may have contributed to the greater height and biomass for inoculated plants than the noninoculated ones due to a better host plant response to AMF colonization attributable to enhanced phosphorus uptake. [24] also noted that plant and AMF attraction to each others' physical interaction is a characteristic plant signal most abundant in phosphate-deprived plant root exudates. Similar results were reported for Acacia species and Eucalyptus species in disturbed soils in Australia and other several researches [41–45]. In addition, dry weight of Acacia and Eucalyptus species [46] was also increased by at least 2-to-3-fold by AMF inoculation under glass house conditions as also observed in this study. AMF populations may also play an important role in natural ecosystems or revegetation, in maintaining plant species diversity, by boosting the ability of mycorrhizal plants to compete for resources [45]. However, all AM fungi contribute differently to nutrient uptake and plant growth as also noted by [41].

## 5. Conclusion and Recommendations

AMF inoculation induced a positive response, and Calliandra responded variably in greenhouse pot experiments and the greater the rate of AMF inoculum application, the better the performance. This suggests that AMF may play an important role in the rapid growth Calliandra plants on forest and agricultural land. Furthermore, significant variation in the effectiveness of different AMF species and at varying rates was demonstrated, with the single AMF isolates (*Glomus* sp.) performing better in Calliandra potted experiment but the mixture showing better results in terms of biomass.

Supplementary work is needed to investigate the effectiveness of single and different combinations of AMF species and at different inoculum application rates with different host plants for enhanced growth of both forest trees and agricultural plants in subsequent degraded lands facilitating their restoration. This is because any such increase in plant or seedling growth as a result of mycorrhizal inoculation carries a corresponding increase in carbon sequestration. This in turn provides a good scope for commercially utilizing the efficient strains of AM fungi for beneficial effects and even with other beneficial rhizosphere microbes in the primary establishment of slow growing seedlings ensuring better survival and improved growth.

On the other hand, inoculation with AMF is a challenging exercise. The technology for producing and distributing inoculum in large quantities is currently poorly developed making it difficult to investigate AMF inoculation in the field. Given the constraints to inoculation, greater attention must be paid to managing the indigenous organisms, which represent a mix of climatically adapted fungi, evolved in association with the host plants to be grown. Effective management would require greater understanding of their ecology and biology, which in turn may allow general predictions of the survival of the fungi after soil disturbance and of the capacity to benefit plant growth. Therefore, further research is required to develop a practical technique for large-scale inoculation with these fungi. As such, an adequate population of AMF, and even in combination with other symbiotic microorganisms, will enhance the long-term sustainability of revegetated ecosystems and/or restoration of degraded lands (such as forests and agricultural lands).

## Figures and Tables

**Table 1:** Relative field mycorrhizal dependency for Calliandra.

Application	30 spores	50 spores
Mixture	9.32	29.66
*Glomus* sp.	27.15	13.39
*Scutellospora* sp.	7.59	23.06
*Gigaspora* sp.	26.38	14.99
*Acaulospora* sp.	0.55	8.18

Average	14.20	17.86
